# Prediction model for postoperative atrial fibrillation in non-cardiac surgery using machine learning

**DOI:** 10.3389/fmed.2022.983330

**Published:** 2023-01-10

**Authors:** Ah Ran Oh, Jungchan Park, Seo Jeong Shin, Byungjin Choi, Jong-Hwan Lee, Kwangmo Yang, Ha Yeon Kim, Ji Dong Sung, Seung-Hwa Lee

**Affiliations:** ^1^Samsung Medical Center, Department of Anesthesiology and Pain Medicine, School of Medicine, Sungkyunkwan University, Seoul, Republic of Korea; ^2^Department of Anesthesiology and Pain Medicine, Kangwon National University Hospital, Chuncheon, Republic of Korea; ^3^Department of Biomedical Sciences, Graduate School of Medicine, Ajou University, Suwon, Republic of Korea; ^4^Institute for Innovation in Digital Healthcare, Yonsei University, Seoul, Republic of Korea; ^5^Center for Health Promotion, Samsung Medical Center, School of Medicine, Sungkyunkwan University, Seoul, Republic of Korea; ^6^Department of Anesthesiology and Pain Medicine, School of Medicine, Ajou University, Suwon, Republic of Korea; ^7^Rehabilitation and Prevention Center, Samsung Medical Center, School of Medicine, Heart Vascular Stroke Institute, Sungkyunkwan University, Seoul, Republic of Korea; ^8^Department of Biomedical Engineering, Seoul National University College of Medicine, Seoul, Republic of Korea

**Keywords:** cardiac event, atrial fibrillation, non-cardiac surgery, machine learning, prediction model

## Abstract

Some patients with postoperative atrial fibrillation (POAF) after non-cardiac surgery need treatment, and a predictive model for these patients is clinically useful. Here, we developed a predictive model for POAF in non-cardiac surgery based on machine learning techniques. In a total of 201,864 patients who underwent non-cardiac surgery between January 2011 and June 2019 at our institution, 5,725 (2.8%) were treated for POAF. We used machine learning with an extreme gradient boosting algorithm to evaluate the effects of variables on POAF. Using the top five variables from this algorithm, we generated a predictive model for POAF and conducted an external validation. The top five variables selected for the POAF model were age, lung operation, operation duration, history of coronary artery disease, and hypertension. The optimal threshold of probability in this model was estimated to be 0.1, and the area under the receiver operating characteristic (AUROC) curve was 0.80 with a 95% confidence interval of 0.78–0.81. Accuracy of the model using the estimated threshold was 0.95, with sensitivity and specificity values of 0.28 and 0.97, respectively. In an external validation, the AUROC was 0.80 (0.78–0.81). The working predictive model for POAF requiring treatment in non-cardiac surgery based on machine learning techniques is provided online (https://sjshin.shinyapps.io/afib_predictor_0913/). The model needs further verification among other populations.

## Introduction

Postoperative atrial fibrillation (POAF) is defined as new onset atrial fibrillation following surgery in patients without prior history of atrial fibrillation (1). PAOF is the most common complication following cardiac surgery and is associated with increased morbidity, mortality, length of hospital stay, and long-term risk of stroke (2, 3). In non-cardiac surgery where the heart is not manipulated directly, the incidence of POAF is reported to be lower and to vary by surgery type (4, 5). Although the majority of POAF converts spontaneously to sinus rhythm, POAF in non-cardiac surgery is known to affect long-term consequences (6). Moreover, current guidelines indicate that a certain portion of POAF patients need immediate medication treatment for heart rate or rhythm control (1, 4, 7). Considering the large number of patients who undergo non-cardiac surgery and their risks as they age, prediction of these events would be helpful in daily clinical practice (8).

Previous studies have attempted to identify risk factors for POAF in non-cardiac surgery (4, 5, 9). Reported risk factors include age, male sex, history of cardiovascular disease, and preoperative heart rate (10). There are limitations in applying individual risk factors in clinical practice because existing studies on these risk factors are heterogeneous and show inconsistent results. Moreover, these studies have been conducted with small numbers of patients or in specific groups with select surgical procedures or diagnoses (4, 5, 9). Here, we aimed to investigate risk factors of non-cardiac surgery in a comprehensive manner and to generate a predictive model of POAF that can be used in clinical practice. We used a large real-world data set of consecutive adult patients and identified those who needed interventional treatment for POAF during the first postoperative 30 days. Based on machine learning techniques, we developed a predictive model that can be applied conveniently in clinical practice and conducted an external validation. For further verification, we provided the model online.

## Materials and methods

Approval for this study was waived by the Institutional Review Board of Samsung Medical Center (SMC 2021-06-078) because the study registry was curated in a de-identified form. The requirement for written informed consent from participants was also waived. Using data for external validation was approved by the Institutional Review Board of Ajou University Hospital (AJIRB-MED-MDB-21-662). The validation cohort was also curated in a de-identified form, so written informed consent was waived. We followed the Declaration of Helsinki and reported according to the guidelines for Strengthening the Reporting of Observational Studies in Epidemiology.

### Data curation and study population

We utilized data from the Samsung Medical Center-Non-Cardiac operation (SMC-NoCop) registry (KCT 0006363), a single-center de-identified cohort of 203,787 consecutive adult patients who underwent non-cardiac surgery under general or regional anesthesia at Samsung Medical Center, Seoul, Korea, between January 2011 and June 2019. The registry is based on the institutional electronic archive system, which contains medical information from electronic hospital records of over 4 million patients with more than 900 million laboratory findings and 200 million prescriptions. Raw data were extracted using “Clinical Data Warehouse Darwin-C,” an electronic system for investigators to search and retrieve de-identified medical records. In this system, mortality is updated consistently and confirmed with the National Population Registry of the Korea National Statistical Office using a unique personal identification number for mortalities following hospital discharge.

After obtaining a preoperative evaluation sheet, investigators independent from this study organized relevant preoperative variables including demographic data, underlying diseases, and information from blood laboratory tests. We also estimated preoperative Charlson Comorbidity Index for patients using preoperative diagnoses based on International Classification of Diseases-10 (ICD-10) codes (11). Postoperative diagnoses were organized based on in-hospital progress notes, nursing charts, discharge notes, results of examinations, and drug prescriptions. For analysis, we excluded patients who experienced preoperative atrial fibrillation.

For external validation, we extracted data from patient records who underwent non-cardiac surgery at Ajou University Medical Center between January 2011 and October 2021. Through the same recruitment criteria, 91,576 patients were included in the external validation set.

### Definitions and study endpoints

Risks in surgical procedures were stratified following the European Society of Cardiology (ESC)/European Society of Anesthesiology (ESA) guidelines on non-cardiac surgery (12). For the predictive model, we included newly-developed POAF events within 30 days following surgery and requiring interventions such as intravenous administration of antiarrhythmic agents such as propafenone, flecainide, amiodarone, diltiazem, or verapamil. We also included patients who required electrical cardioversion for rhythm or rate control.

The primary endpoint was POAF requiring interventional treatment during hospital stay within 30 days after non-cardiac surgery. From a total of 201,864 patients, newly developed POAF occurred in 7,757 (3.8%), and 5,725 (2.8%) required interventional treatment. We quantified and compared the effects of each variable on the predictive performance of the model. After conducting feature elimination, we developed a calculator for POAF prediction.

### Development of the predictive model

A total of 50 predictor variables obtained from a preoperative evaluation sheet was provided as input to each model (Supplementary Table 1). We applied machine learning techniques with an extreme gradient boosting (XGB) algorithm, which is a decision tree-based ensemble model using a gradient boosting framework and the Shapley value framework (13, 14). The hyper-parameters were optimized based on a grid search using the area under the receiver operating characteristic (AUROC) curve, and 5-fold cross-validation was employed during model development. We divided the data into training and test sets. A stratified random split of the data was conducted while maintaining a constant ratio of an event, POAF in this study, and 80% of the data were reserved for creating the machine learning model, and the remaining 20% was for the testing model.

Feature interpretation was presented in a SHapley Additive exPlanations (SHAP) summary plot. The impact of each feature on POAF was presented as a SHAP value, which represents the characteristic of deriving a marginal distribution and weighted average by fixing all variables except one and predicting that one to determine its importance (14). In the SHAP summary plot, features are sorted in descending order by effect on POAF, and each patient is represented by one dot on each variable line. The horizontal location of each dot indicates the level of association between the feature and outcome. The area shown on the right side is the point where the SHAP value is greater than zero. Variable-specific SHAP values >0 indicate increased risk.

For practical use in clinical practice, we eliminated variables and developed a predictive model for POAF with the fewest number of variables. We also leveraged Shiny, an application-building package from R, which users can access gratis *via* a public link. Our model was developed based on our observed top five patient features. Using an estimated threshold for probability, AUROC, accuracy, sensitivity, and specificity were computed. For further validation, we generated a case-balanced dataset within an internal dataset and also conducted an external validation.

### Statistical analysis

We compared differences between patients who developed POAF following non-cardiac surgery and patients who did not. Continuous features are presented as mean ± standard deviation or median with interquartile range, and comparisons were conducted by *t*-test or Mann-Whitney test, as applicable. Categorical features are presented as number and percentage, and differences were evaluated using Chi-square or Fisher’s exact test. Analysis was performed using R 4.1.0 (Vienna, Austria)^1^.

## Results

### Baseline characteristics and mortality

We excluded 1,923 patients with preoperative atrial fibrillation. The baseline characteristics of patients with and without POAF are presented in Table 1. Patients with POAF were older, predominantly male, and had a higher incidence of underlying disease. In patients with POAF, preoperative hemoglobin level was significantly lower and creatinine level was higher. Large differences in operative variables were observed between patients with and without POAF (Table 2).

**TABLE 1:** Preoperative variables in patients with and without postoperative atrial fibrillation.

	No atrial fibrillation (*N* = 196,139)	Atrial fibrillation (*N* = 5,725)	*P*-value
Male	83,392 (42.5)	3,512 (61.3)	<0.001
Age	52.3 (±15.1)	64.1 (±12.5)	<0.001
Hypertension	48,033 (24.5)	2,645 (46.2)	<0.001
Diabetes	21,819 (11.1)	1,324 (23.1)	<0.001
Current alcohol	39,590 (20.2)	704 (12.3)	<0.001
Current smoking	15,203 (7.8)	310 (5.4)	<0.001
Chronic kidney disease	3,040 (1.5)	236 (4.1)	<0.001
Dialysis	866 (0.4)	68 (1.2)	–
**Previous disease**			
Charlson comorbidity index	0.1 (±0.5)	0.4 (±1.1)	<0.001
Stroke	3,812 (1.9)	309 (5.4)	<0.001
Coronary artery disease	3,181 (1.6)	821 (14.3)	<0.001
Myocardial infarction	809 (0.4)	89 (1.6)	<0.001
**Coronary revascularization**			
Percutaneous intervention	2,510 (1.3)	439 (7.7)	<0.001
Bypass graft	354 (0.2)	58 (1.0)	<0.001
Heart failure	380 (0.2)	108 (1.9)	<0.001
Arrhythmia	906 (0.5)	184 (3.2)	<0.001
Peripheral artery disease	508 (0.3)	45 (0.8)	<0.001
Aortic disease	603 (0.3)	80 (1.4)	<0.001
Valvular heart disease	211 (0.1)	35 (0.6)	<0.001
Chronic obstructive pulmonary disease	3,216 (1.6)	307 (5.4)	<0.001
**Preoperative blood laboratory tests**			
Hemoglobin, g/dl	13.3 (±1.8)	12.9 (±2.0)	<0.001
Creatinine, mg/dl	0.9 (±0.8)	1.0 (±1.0)	<0.001
**Preoperative vital signs**			
Heart rate, bpm	68.2 (±11.6)	71.0 (±15.2)	<0.001
Systolic blood pressure, mmHg	119.2 (±16.6)	122.6 (±17.9)	<0.001
Diastolic blood pressure, mmHg	70.7 (±10.9)	71.6 (±11.1)	<0.001
Mean blood pressure, mmHg	86.9 (±11.7)	88.6 (±12.0)	<0.001

Data are presented as *n* (%) or mean (±standard deviation).

**TABLE 2:** Operative variables in patients with and without postoperative atrial fibrillation.

	No atrial fibrillation (*N* = 196,139)	Atrial fibrillation (*N* = 5,725)	*P*-value
General anesthesia	169,710 (86.5)	5,170 (90.3)	<0.001
Emergency operation	13,602 (6.9)	629 (11.0)	<0.001
Operation duration, min	130.8 (±100.3)	170.8 (± 118.9)	<0.001
Surgical risk	–	–	<0.001
Mild	77,427 (39.5)	1,280 (22.4)	–
Intermediate	107,282 (54.7)	3,518 (61.4)	–
High	11,430 (5.8)	927 (16.2)	–
Inotropic drug infusion	15,762 (8.0)	1,534 (26.8)	<0.001
Blood transfusion	6,321 (3.2)	560 (9.8)	<0.001
Red blood cell	6,129 (3.1)	531 (9.3)	<0.001
Cryoprecipitate	365 (0.2)	40 (0.7)	<0.001
Fresh frozen plasma	1,262 (0.6)	151 (2.6)	<0.001
Platelet concentrate	229 (0.1)	37 (0.6)	<0.001
Surgery types	–	–	–
Neuroendocrine	12,889 (6.6)	136 (2.4)	<0.001
Lung	10,682 (5.4)	1,277 (22.3)	<0.001
Head and neck	30,129 (15.4)	702 (12.3)	<0.001
Breast	17,477 (8.9)	159 (2.8)	<0.001
Stomach	12,200 (6.2)	279 (4.9)	<0.001
Hepatobiliary	16,566 (8.4)	485 (8.5)	0.97
Colorectal	13,331 (6.8)	409 (7.1)	0.32
Urology	17,942 (9.1)	445 (7.8)	<0.001
Gynecology	24,348 (12.4)	156 (2.7)	<0.001
Bone and skin etc.	40,575 (20.7)	1,677 (29.3)	<0.001

Data are presented as *n* (%) or mean (±standard deviation). Surgical risk was stratified according to the 2014 European Society of Cardiology (ESC)/European Society of Anesthesiology (ESA) guidelines.

### Predictive models for POAF

**FIGURE 1:**
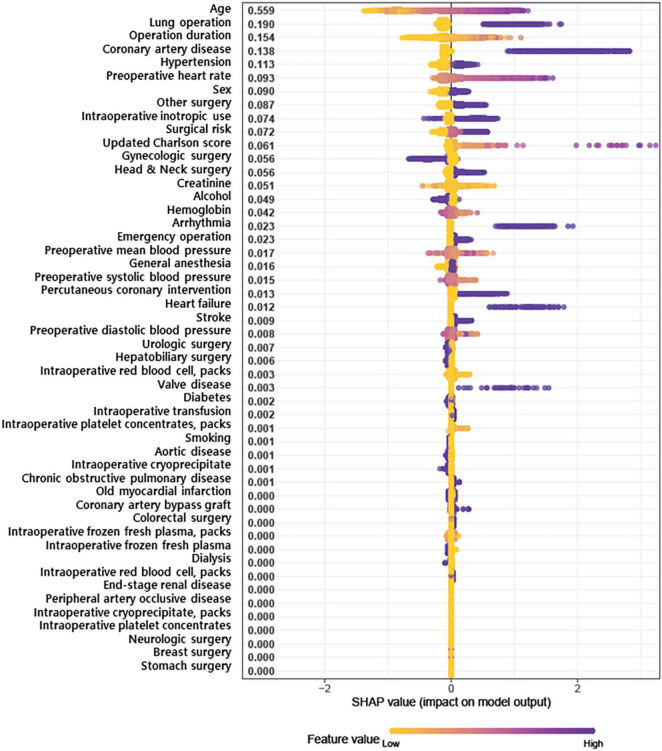
SHapley Additive exPlanations (SHAP) summary plot representing the results of a machine learning-based extreme gradient boosting (XGB) algorithm.

The SHAP summary plot for results of the XGB model is shown in Figure 1, with features shown in descending order of contribution to PAOF development. The horizontal line comprised of dots presents variable effects on POAF. Representing patient characteristics, SHAP values greater than zero (presented on the right side) indicate increased risk, while values on the left side indicate lower risk for POAF. The top five variables with SHAP values greater than 0.1 were age (0.559), lung operation (0.190), operation duration (0.154), history of coronary artery disease (0.138), and hypertension (0.113).

**FIGURE 2:**
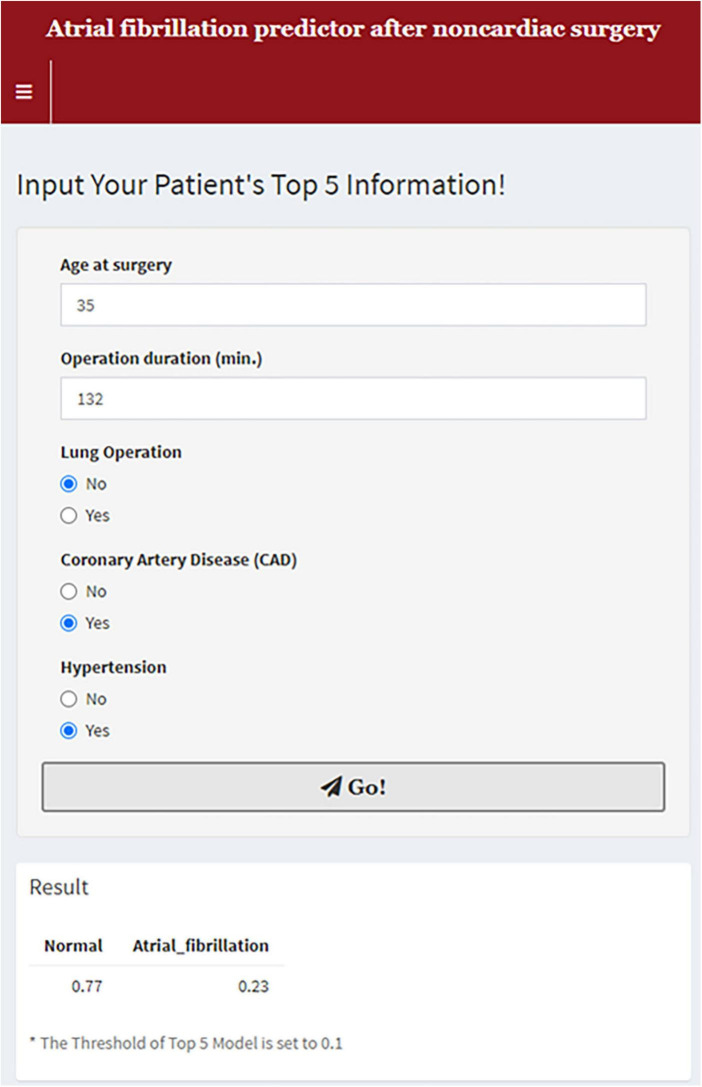
An online predictive model for postoperative atrial fibrillation.

**FIGURE 3:**
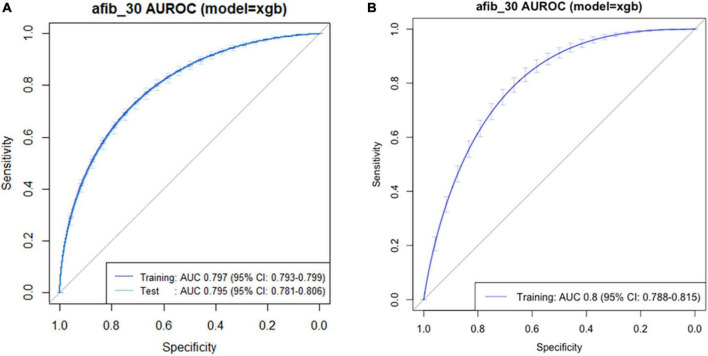
Receiver operating characteristic curve of the predictive model for postoperative atrial fibrillation in **(A)** original and **(B)** external validation datasets.

To apply our findings to clinical practice, we eliminated a number of variables in the predictive model, as the predictive models based on 10 and seven variables showed similar power (Supplementary Figure 1). Finally, we developed the predictive model based on the top five variables, which is simpler and more convenient for clinical use. A functioning version of the model is provided online at https://sjshin.shinyapps.io/afib_predictor_0913/ (Figure 2). When values for each of the top five variables for target patients are entered, the probability for POAF is shown as an output. An optimal threshold of probability in this model was estimated based on the maximal Youden index (Supplementary Table 2). We also estimated the correlation matrix of the selected features (Supplementary Figure 2). They were all below 0.2 except for the one between hypertension and age, indicating that there was a correlation only between hypertension and age among the top five variables. The receiver operating characteristic curve of the model is shown in Figure 3. Applying 0.1 as a threshold, the AUROC was 0.80 with a 95% confidence interval of 0.78–0.81. Accuracy of this threshold was 0.95, with sensitivity and specificity values of 0.28 and 0.97, respectively. The F1 score was 0.222, and precision was 0.185. In a case-balanced dataset, the AUROC was 0.77 with sensitivity and specificity values of 0.85 and 0.68, respectively. The F1 score was 0.785, and precision was 0.727.

### External validation of the predictive model

The external validation dataset consisted of 91,576 patients. POAF developed in 1,977 (2.2%) patients, and 790 (0.9%) required interventional treatments. Based on the top five variables, our predictive model achieved an AUROC of 0.80 (0.78–0.81) in an external validation dataset (Figure 3). Using the same threshold of 0.1, the sensitivity and specificity values were 0.21 and 0.89, respectively.

## Discussion

In this study, we used machine learning techniques with an XGB algorithm to identify variables associated with POAF requiring treatment in non-cardiac surgery and created a predictive model. The incidence of POAF was 2.8%, and the top five variables retained in our predictive model were age, lung operation, operation duration, history of coronary artery disease, and hypertension. Our predictive model achieved an AUROC value of 0.80 (95% confidence interval 0.78–0.81) at a threshold of 0.1.

Postoperative atrial fibrillation in non-cardiac surgery is typically considered to be a transient and reversible phenomenon of minor clinical significance. However, recent studies have revealed that POAF in non-cardiac surgery is associated with increased risk of long-term complications such as ischemic stroke (15–17). In addition, some POAF patients demonstrate instability in vital signs. Immediate treatment to control heart rate or rhythm can be urgent for these patients. In this regard, a predictive model for POAF requiring interventional treatment can be useful in postoperative care. Accordingly, we aimed to develop a predictive model that can be used in non-cardiac surgery to identify potentially high-risk patients and to adjust care and treatment at the individual level.

When applying artificial intelligence such as machine learning techniques in a field of medicine, interpretability should be primarily considered (18). We chose variables based on SHAP feature importance, and those that were clinically explainable. Age, which ranked as the first variable, has consistently been reported as a predictor of POAF in previous studies (4, 5, 9). This association is well-explained by age-associated structural changes and fibrosis in the atrium that provide substrate for arrhythmias (19). Another explanation could be the higher incidence of cardiovascular comorbidities in older patients. Comorbidities such as hypertension and coronary artery disease are also used in our predictive model. In hypertensive patients, the renin angiotensin aldosterone system and sympathetic outflow are activated, resulting in left ventricular hypertrophy, diastolic dysfunction, and atrial stiffness—all of which induce atrial fibrillation (20). Coronary artery disease contributes to POAF with a bidirectional relationship (21). The pro-inflammatory condition of coronary artery disease can cause myocardial inflammation exacerbated by a supply-demand mismatch, which triggers atrial fibrillation by altering cardiac conduction (22). In addition, myocardial infarction leads to left ventricular remodeling that can predispose patients to atrial fibrillation. Similar processes can occur in hypoxic patients, and coronary artery disease might lower the threshold for POAF development (10).

Among operative variables, thoracic surgery is well-known to result in a high incidence of POAF, ranging from 6.4 to 19% in various patient samples (23, 24). The atrial stretching induced by pulmonary vasoconstriction and local inflammation of pulmonary veins can contribute to POAF (10). In thoracic surgery, aspects of the procedure are known to affect the incidence of POAF (24). According to our SHAP summary plot, a short operation time had a definite negative impact on POAF, but a long operation time did not have a clear positive impact. Numerous studies have demonstrated that operation duration is associated with postoperative outcomes (25), but an association with POAF has rarely been reported. This might be due to limitations in traditional regression models that implicitly assume a linear relationship between each risk factor and outcomes (26).

In the present study, XGB algorithms were used for machine learning techniques to evaluate the broad spectrum of non-cardiac surgical procedures with various diagnoses. XGB algorithms are known for superior performance in comparison to traditional algorithms and have been used to develop effective prediction models in a wide range of applications (13). We relied on SHAP feature importance based on Shapley values because they are computationally fast and have good theoretical properties (14). A Shapley value is defined as the average marginal contribution of a feature across all possible feature coalitions. Under this definition, Shapley values are affected by incidence, and the positions of dots on the y-axis should be considered in interpreting SHAP summary plots. Our results show the relatively low importance of valvular heart disease, which is a well-known risk factor for POAF (27), and this might be related to the low prevalence of valvular heart disease in our cohort. Traditional methods can ignore complex relationships among a large number of risk factors with non-linear interactions (26). By applying an advanced model that considers multiple risk factors, we aimed to overcome the conventional drawbacks of traditional statistical models.

An additional strength is that our predictive model can be applied widely. It was based on heterogeneous patients undergoing a broad spectrum of surgical procedures, and the generated model was further validated with an external dataset. In the external validation, our model achieved similar predictive power, despite lower incidence of POAF. The incidence of POAF has been reported to vary widely in previous studies, and our results suggest that our model can be applied in groups of patients with different characteristics. Furthermore, our predictive model is available to the public for further verification or clinical application.

### Study strengths and limitations

There are several study limitations that need to be considered. First, due to the nature of the retrospective data, causality cannot be confirmed in the current study. Moreover, we could not evaluate the difference between self-limited POAF and POAF requiring intervention due to the nature of the dataset. The presence of underlying disease as variables was all self-reported. Second, perioperative care was not controlled during the long period of study. Despite the presence of an institutional protocol based on current guidelines, decisions were often made at the discretion of attending clinicians. Third, we cannot generalize our results to other patient groups due to the limitations of a single-center study, and ethnic differences were not considered. In addition, our model could not be compared with pre-existing predictive models for POAF in cardiac surgery because our study aimed to develop a predictive model in non-cardiac surgery. Detailed preoperative cardiac evaluations, including findings such as left ventricular ejection fraction or coronary artery angiograms, were not available for all patients in our sample. Fourth, the causality-based feature selection is not guaranteed in our model. To be specific, SHAP values are a measure of the importance of a feature related to the model which is different from the importance of a feature for the outcome. Last, factors retained in our model were mostly non-modifiable, and additional prevention or treatment strategies could not be established. Despite these limitations, this is the first study to identify risk factors for POAF in non-cardiac surgery using a machine learning algorithm and a proven predictive model.

## Conclusion

Using five variables identified by machine learning techniques, we developed a predictive model for POAF requiring treatment with good predictive power in patients undergoing non-cardiac surgery. The predictive model is available online, with the model in need of further verification among other populations.

## Data availability statement

The raw data supporting the conclusions of this article will be made available by the authors, without undue reservation.

## Ethics statement

Approval for this study was waived by the Institutional Review Board of Samsung Medical Center (SMC 2021-06-078) because the study registry was curated in a de-identified form. The requirement for written informed consent from participants was also waived. Using data for an external validation was approved by the Institutional Review Board of Ajou University Hospital (AJIRB-MED-MDB-21-87 662). The validation cohort was also curated in a de-identified form, so the written informed consent was waived.

## Author contributions

S-HL: conceptualization. AO, JP, and HK: data curation. SS and BC: formal analysis. J-HL, KY, and JS: supervision. AO and JP: first draft. All authors contributed to article, revision of manuscript, and approved the submitted version.

## Footnotes

1
http://www.R-project.org/
