# Supplementary figures and images for: Prediction model for postoperative atrial fibrillation in non-cardiac surgery using machine learning
Source: Front Med (Lausanne). 2023 Jan 10;9:983330. doi: 10.3389/fmed.2022.983330 (PMC9871538; doi:10.3389/fmed.2022.983330)

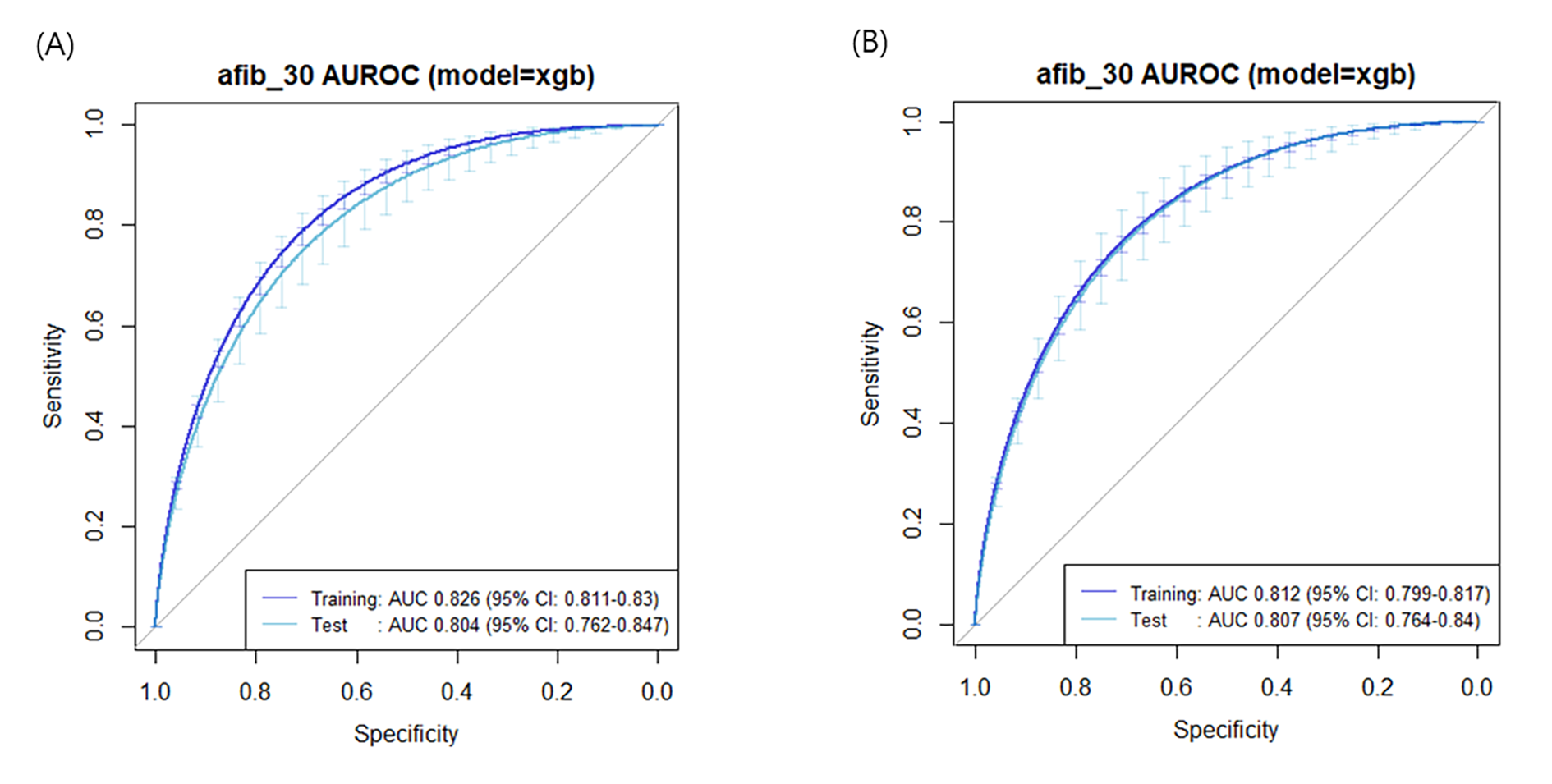

Supplement: Supplementary file 1 [file Image_1.TIF]

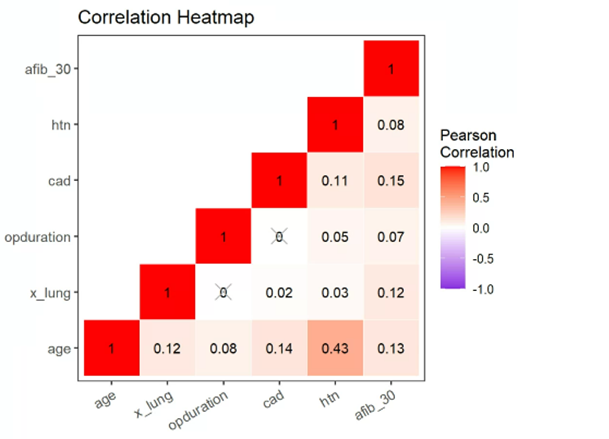

Supplement: Supplementary file 2 [file Image_2.TIF]
