# Supplementary material for: Prediction model for postoperative atrial fibrillation in non-cardiac surgery using machine learning
Source: Front Med (Lausanne). 2023 Jan 10;9:983330. doi: 10.3389/fmed.2022.983330 (PMC9871538; doi:10.3389/fmed.2022.983330)
Supplement: Supplementary file 3 [file Table_1.DOCX]

Supplemental table 1. Variables

| Demographic data | Male |
| --- | --- |
|  | Age* |
|  | Hypertension |
| Physical function and underlying disease | Diabetes |
| Self-reported by patients | Current alcohol |
|  | Current smoking |
|  | Chronic kidney disease |
|  | Dialysis |
|  | Charlson comorbidity index |
|  | Stroke |
|  | Coronary artery disease |
|  | Coronary revascularization |
|  | Percutaneous intervention |
|  | Bypass graft |
|  | Heart failure |
|  | Arrhythmia |
|  | Atrial fibrillation |
|  | Peripheral artery disease |
|  | Aortic disease |
|  | Valvular heart disease |
|  | Chronic obstructive pulmonary disease |
| Preoperative blood laboratory tests and vital signs | Hemoglobin, g/dl* |
|  | Creatinine, mg/dL* |
|  | Preoperative vital signs |
|  | Heart rate, bpm* |
|  | Systolic blood pressure, mmHg* |
|  | Diastolic blood pressure, mmHg* |
|  | Mean blood pressure, mmHg* |
| Operative factors | General anesthesia |
|  | Emergency operation |
|  | Operation duration, min* |
|  | Surgical risk |
|  | Mild |
|  | Intermediate |
|  | High |
|  | Surgery types |
|  | Neuroendocrine |
|  | Lung |
|  | Head & Neck |
|  | Breast |
|  | Stomach |
|  | Hepatobiliary |
|  | Colorectal |
|  | Urology |
|  | Gynecology |
|  | Bone & Skin etc |

* analyzed as continuous variable

Supplemental table 2. Predictability according to thresholds

| Threshold | Accuracy | Sensitivity | Specificity | Positive predictive value | Negative predictive value |
| --- | --- | --- | --- | --- | --- |
| 0 | 0.028 | 1 | 0 | 0.028 | NA |
| 0.01 | 0.028 | 1 | 0 | 0.028 | NA |
| 0.02 | 0.028 | 1 | 0 | 0.028 | NA |
| 0.03 | 0.898 | 0.395 | 0.912 | 0.116 | 0.981 |
| 0.04 | 0.898 | 0.394 | 0.913 | 0.116 | 0.981 |
| 0.05 | 0.898 | 0.394 | 0.913 | 0.116 | 0.981 |
| 0.06 | 0.9 | 0.39 | 0.914 | 0.118 | 0.981 |
| 0.07 | 0.9 | 0.39 | 0.915 | 0.118 | 0.981 |
| 0.08 | 0.9 | 0.39 | 0.915 | 0.118 | 0.981 |
| 0.09 | 0.946 | 0.219 | 0.967 | 0.161 | 0.977 |
| 0.1 | 0.951 | 0.197 | 0.973 | 0.175 | 0.976 |
| 0.11 | 0.953 | 0.185 | 0.975 | 0.179 | 0.976 |
| 0.12 | 0.954 | 0.183 | 0.976 | 0.183 | 0.976 |
| 0.13 | 0.957 | 0.161 | 0.981 | 0.196 | 0.976 |
| 0.14 | 0.958 | 0.158 | 0.982 | 0.2 | 0.976 |
| 0.15 | 0.958 | 0.158 | 0.982 | 0.202 | 0.976 |
| 0.16 | 0.963 | 0.125 | 0.988 | 0.227 | 0.975 |
| 0.17 | 0.963 | 0.124 | 0.988 | 0.226 | 0.975 |
| 0.18 | 0.963 | 0.12 | 0.988 | 0.221 | 0.975 |
| 0.19 | 0.969 | 0.048 | 0.995 | 0.237 | 0.973 |
| 0.2 | 0.97 | 0.04 | 0.997 | 0.288 | 0.973 |
